# Source Tracing of *Leishmania donovani* in Emerging Foci of Visceral Leishmaniasis, Western Nepal

**DOI:** 10.3201/eid3003.231160

**Published:** 2024-03

**Authors:** Pieter Monsieurs, Kristien Cloots, Surendra Uranw, Megha Raj Banjara, Prakash Ghimire, Sakib Burza, Epco Hasker, Jean-Claude Dujardin, Malgorzata Anna Domagalska

**Affiliations:** Institute of Tropical Medicine, Antwerp, Belgium (P. Monsieurs, K. Cloots, E. Hasker, J.-C. Dujardin, M.A. Domagalska);; BP Koirala Institute of Health Sciences, Dharan, Nepal (S. Uranw);; Tribhuvan University, Kathmandu, Nepal (M.R. Banjara, P. Ghimire);; London School of Hygiene and Tropical Medicine, London, UK (S. Burza)

**Keywords:** *Leishmania donovani*, leishmaniasis, West Nepal, Indian subcontinent, parasites

## Abstract

We sequenced *Leishmania donovani* genomes in blood samples collected in emerging foci of visceral leishmaniasis in western Nepal. We detected lineages very different from the preelimination main parasite population, including a new lineage and a rare one previously reported in eastern Nepal. Our findings underscore the need for genomic surveillance.

*Leishmania* spp. are parasitic protozoans that cause human leishmaniasis in multiple forms, including visceral leishmaniasis (VL), which affects the internal organs. For decades, the Indian subcontinent (ISC)—a geographic region that includes Bangladesh, Bhutan, India, Maldives, Nepal, Pakistan, and Sri Lanka—was the most endemic region for VL in the world. In 2005, a regional elimination program was launched in India, Nepal, and Bangladesh, aiming to reduce VL annual incidence to <1 case/10,000 population at subdistrict and district levels (*1*). Before the start of the program, VL in Nepal was confined mainly to 12 VL endemic districts (out of 77), located in the eastern lowlands. Recently, VL cases in Nepal have spread westward, as well as from lowlands to hilly and even mountainous areas, resulting in a current total of 23 official VL endemic districts, with many more districts reporting likely indigenous cases (*1*). Cutaneous leishmaniasis is also becoming more common (*2*), and combined cases of VL and cutaneous leishmaniasis have been reported, without any information to date on the parasite species and genotype involved. There is clearly a need for a postelimination surveillance system adapted to this new epidemiologic profile.

Molecular surveillance of infectious diseases may provide the most relevant information for control programs, such as following the evolution of epidemics in time and space, characterizing of new transmission cycles, conducting outbreak studies and source identification, and detecting new variants with new clinical features (*3*). Currently, no molecular surveillance is being implemented for leishmaniasis in the world, despite the existence of suitable technologies. We previously showed the feasibility and added value of direct whole genome sequencing (SureSelect sequencing [SuSL-seq]; Agilent Technologies, https://www.agilent.com) of *L. donovani* in host tissues, without the need for parasite isolation and cultivation (*4*).

Here, we demonstrate the proof-of-principle of SuSL-seq for genome surveillance of leishmaniasis, in the context of the reported expansion of VL to the western regions of Nepal. We collected blood samples in 2019 and stored them on DNA/RNA Shield (Appendix). We performed sequencing on 3 samples with the highest amounts of DNA, positive for *Leishmania*, and originating from 3 different districts in Nepal (Dolpa, Darchula, and Bardiya) (Appendix Table 1, Figure 1) and compared them with our database of *L. donovani* genome sequences from the ISC. All samples showed a high genome coverage (Appendix Table 2). The database comparison samples originated from 204 cultivated isolates (2002–2011) from Nepal, India, and Bangladesh (*5*); 52 clinical samples (2000–2015) from Nepal (*4*); and 3 isolates (2002, 2010) from Sri Lanka (*6*,*7*). Altogether, these earlier studies reported 4 main genotypes: a large core group (CG), genomically very homogeneous, in the lowlands of India, Nepal, and Bangladesh; a small ISC1 population, genomically very different from CG, in hilly districts of Eastern Nepal; a single divergent isolate from Nepal, BPK512; and a Sri Lanka (SL) cluster. New phylogenomic analyses (Figure) revealed that the samples from the 3 new foci from western Nepal were clearly distinct from CG and SL: one ISC1-related lineage (024) had not been reported previously, and the 2 other lineages (022 and 023) clustered together with BPK512.

**Figure:**
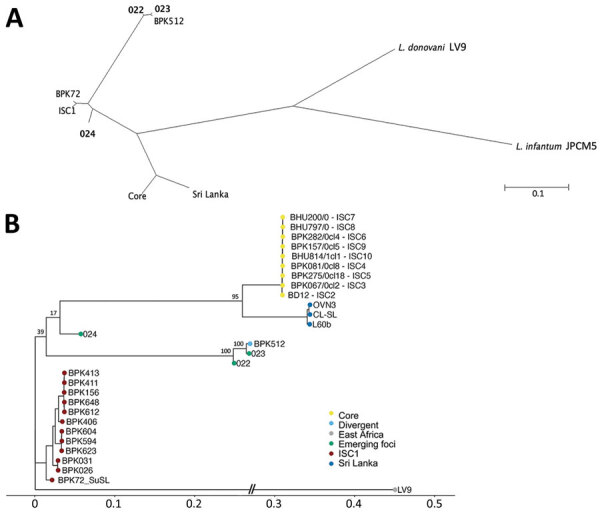
Phylogenetic analyses of *Leishmania donovani* from the ISC, including Nepal, and reference sequences. Trees were based on genomewide single-nucleotide polymorphisms using RAxML (*8*). A) Unrooted phylogenetic network of the *L. donovani* complex, showing samples representing the emerging foci (bold text). B) Rooted phylogenetic tree of reference strains of *L. donovani* from the ISC, showing the branching of 3 samples (022, 023, and 024) originating from emerging foci. Important bootstrap values are indicated on the branches. The West-African LV9 strain is included as an outgroup. BPK72_SuSL represents an ISC1 sample analyzed using SureSelect sequencing (Agilent Technologies; https://www.agilent.com), confirming that the branching of the emerging foci is not a result of a technical artifact. Scale bars indicate number of single-nucleotide polymorphism differences. ISC, Indian subcontinent.

It is premature to conclude that ISC1-related (024) and BPK512-like (022, 023) parasites are expanding, spreading, and replacing CG in a postelimination phase. However, a study based on single-locus genotyping showed a much higher proportion of ISC1 and unclassified genotypes (and a strong decrease of CG) during 2012–2014 compared with 2002–2011 (*9*). Considering the genomic differences between these lineages and CG and their transmissibility by *Phlebotomus argentipes* (*10*), we recommend particular attention to the further evolution of parasites in regions of the ISC. Our previous work evidenced several important functional differences between isolates from ISC1 and CG (Appendix), and we found in this investigation allele differences in 8 of 10 genes previously shown to be involved in *L. donovani* drug resistance (Appendix Figure 2). Of particular interest, those genetic variants are common in the ISC1 group and in the BPK512 but never found in CG parasites. Without experimental confirmation, it is difficult to speculate about the exact impact of this polymorphism on the resistance to antileishmanial drugs, but it is clear that these parasites are genetically (and, likely, functionally) very diverse from the CG parasites, which were the main target of the recent elimination efforts.

Molecular surveillance requires a method applicable on routine samples collected in any type of field settings. We demonstrate that small amounts of blood from routine examination of patients with VL could be successfully used for direct, sensitive, and untargeted whole-genome analysis of *Leishmania*. Our optimized SuSL-seq protocol enables highly discriminatory genotyping and targeted analysis of the genetic variation within selected loci as well as untargeted searching for new markers related to a clinical or epidemiologic question. Our research supports the need for genomic surveillance of VL—in particular in the context of the current elimination program in the ISC—and demonstrate the applicability of SuSL-seq to molecular surveillance of blood. Continued collaborations will be required to translate these new approaches for VL surveillance to the specific needs of the region.

Appendix More information for source tracing of *Leishmania donovani* in emerging foci of visceral leishmaniasis, western Nepal.
