# Supplementary material for: Source Tracing of Leishmania donovani in Emerging Foci of Visceral Leishmaniasis, Western Nepal
Source: Emerg Infect Dis. 2024 Mar;30(3):611–3. doi: 10.3201/eid3003.231160 (PMC10902524; doi:10.3201/eid3003.231160)
Supplement: Appendix — More information for source tracing of Leishmania donovani in emerging foci of visceral leishmaniasis, western Nepal. [file 23-1160-Techapp-s1.pdf]

Article DOI: http://doi.org/10.3201/eid3003.231160

EID cannot ensure accessibility for supplementary materials supplied by authors. Readers who have difficulty accessing supplementary content should contact the authors for assistance.

Source Tracing of *Leishmania donovani* in Emerging Foci of Visceral Leishmaniasis, Western Nepal

Appendix

Ethical clearance, clinical and data procedures

Ethical approval was obtained from the Institutional Review Committee of B.P. Koirala Institute of Health Sciences, Dharan, Nepal, as well as from the Nepal Health Research Council, Kathmandu, Nepal. In addition, ethical approval was obtained by the Institutional Review Board of the Institute of Tropical Medicine, Antwerp, and the Ethics Committee of the University Hospital of Antwerp, Belgium. Collaborating VL treatment centers were asked to collect a 2ml venous blood sample from all consenting newly diagnosed VL patients before the start of any treatment in January and February 2019. VL was diagnosed in line with the clinical algorithm recommended in the National Guidelines, i.e., fever >2 weeks in combination with splenomegaly and a positive rK39 Rapid Diagnostic Test (RDT). In addition, information was collected on basic demographic factors as well as the village of residence and travel history in the 2 years before the start of symptoms (Appendix Table 1, Appendix Figure 1). Samples were collected in DNA/RNA shield and stored at room temperature until transportation to the laboratory facilities of the Central Department of Microbiology at Tribhuvan University, Kathmandu, Nepal, where they were stored at −20°C until DNA extraction. DNA extracts were shipped to the laboratory facilities of the Institute of Tropical Medicine for further sequencing.

Laboratory procedures

DNA extraction and sequencing

200µl of blood mixed with 200µl DNA/RNA Shield was used to extract DNA with QIAamp DNA Mini Kit (Qiagen) according to manufacturer's instructions with the following modifications: 30µl Proteinase K and 300µl ethanol were used instead of 20µl and 200µl, respectively. DNA concentration was verified using the Qubit broad-range DNA quantification kit (Thermo Fisher Scientific), and the % of *Leishmania* DNA in the samples was estimated using qPCR as described previously (*1*). In our previous study (*1*), we demonstrated that a percentage of *Leishmania* DNA of 0.006% was found to be the lowest limit for suitable analysis of genome diversity. In present study, the *Leishmania* % in the selected samples was 0.13, 0.16 and 0.03 in samples 022, 023, and 024, respectively. SureSelect (Agilent Technologies) was used to capture *Leishmania* genomic DNA following standard SureSelect XTHS Target Enrichment system protocol for Illumina Multiplexed Sequencing platforms. Prior the genome capture, DNA was concentrated using AMPure XP beads (Beckman, Coulter) to obtain ≈10ng of total genomic DNA in 7 µl that was subjected to enzymatic fragmentation using SureSelect XT HS Fragmentation kit (Agilent Technologies). Custom designed oligonucleotide baits were used at 1:10 stock dilution.

Sequencing was conducted on the Illumina NovaSeq platform using 2x150 bp paired reads at GenomeScan (Netherlands), for which 51.49, 51.59 and 50.35 million raw reads were obtained for sample 022, 023 and 024 respectively (Appendix Table 2).

Bioinformatic procedures

In addition to the newly sequenced data, additional sequencing data were obtained from previous publications: i) genomes describing the population structure of *L. donovani* in Nepal, India and Bangladesh (*2*), ii) sequencing data obtained from samples from Nepal using the SureSelect technology (*1*), similar to the approach used for the three outbreak samples in this report, iii) three genomes originating from Sri Lanka (*3*,*4*), and iv) the *L. infantum* sequencing data submitted under the accession number ERR1913337 (*5*). All publicly available sequencing data were downloaded using the SRAtoolkit software.

The reads were mapped to the reference genome *L. donovani* available at NCBI (accession number GCF_000227135.1) using BWA (version 0.7.17 (*6*)) with a seed length set to

50 17 (*6*). Only properly paired reads with a mapping quality higher than 30 were selected using SAMtools (*7*). Duplicate reads were removed using the RemoveDuplicates command in the Picard software (version 2.22.4, http://broadinstitute.github.io/picard/). SNP calling was performed using the Genome Analysis ToolKit (GATK) (*8*) pipeline (version 4.1.4.1) following the GATK best practices approach: 1) GATK HaplotypeCaller enabling the production of GVCF formatted files, 2) GVCF files of all samples were combined using the GATK CombineGVCF command, 3) genotyping was performed via the GATK GenotypeGVCF command, and 4) filtering of the SNPs and indels was carried out following the "best practices" approach as suggested on the GATK support site using the SelectVariants and VariantFiltration commands. Regions in the vcf-file corresponding to known drug resistance markers were selected using BCFtools, and visualized using the pheatmap function in R.

Phylogenetic trees were constructed using RAxML (*9*). First, the VCF files containing biallelic SNPs were selected using BCFtools (*7*) and were converted to Phylip format using the vcf2phylip.py script (https://github.com/edgardomortiz/vcf2phylip). RAxML was then executed with the GTR+G substitution model, using 1000 bootstrap replicates. The *L. infantum* JPCM5 or the *L. donovani* LV9 genome was employed as an outgroup. The resulting phylogenetic trees were visualized using ggtree (*10*) for rooted phylogenetic trees and SplitsTree (*11*) for unrooted phylogenetic networks.

Results from the analyses of specific genes reported to be involved in drug resistance

We selected 10 loci that were previously shown to be involved in *L. donovani* resistance to known antileishmanial drugs:

- Antimony: Aquaglyceroporin 1, AQP1 LDBPK_310030 (*12*); ABC transporter MRPA, MRPA LDBPK_230290 (*13*)
- Amphotericin B: sterol C5-desaturase, C5D LDBPK_231560 (*14*); sterol C24-methyltransferase, SMT LDBPK_362520 (*14*)
- Miltefosine: *Leishmania donovani* miltefosine transporter, LdMT LDBPK_131590 (*15*); Beta-subunit of LdMT, LdRos3 LdBPK_320540 (*15*) and genes part of miltefosine sensitivity locus (*16*): 3,2-trans-enoyl-CoA isomerase 1 and 2, TECI1

LdBPK_312320 and TECI2 LdBPK_312400; helicase-like protein, HELI LdBPK_312390; 3′-nucleotidase/nuclease, NUC LdBPK_312380.

For each of the three new *L. donovani* genomes, the sequence of the 10 loci was studied in detail. The 10 loci were well-covered and in 8 out of the 10 genes we found at least one homozygous single nucleotide polymorphism (SNP), which results in a missense mutation or a frameshift in at least 2 out of 3 samples from new emerging loci (Appendix Figure 2). No significant changes were observed for LdRos3 and SMT.

Functional differences previously reported between ISC1 and CG isolates

Noteworthy, all results here compiled concern the analysis of isolated and cultivated parasites.

In a first study, we demonstrated that CG parasites were intrinsically more tolerant to trivalent antimonials than ISC1 ones. This phenotype was driven by the amplification of a locus containing MRPA, a gene involved in Sb^{III} sequestration (*13*)

In a second study, we made an integrated genomic and metabolomic profiling of ISC1 vs CG isolates (*17*). We found several genomic differences including SNPs, CNV and small indels in genes coding for known virulence factors, immunogens and surface proteins. With respect to the metabolome, we found differences in several functional groups and pathways, essentially:

(i) **Lipid metabolism**, with 19 glycerophospholipids (GPLs) showing significantly different levels between both groups: GPLs are involved in a wide array of cellular functions including host cell infection

(ii) **Urea cycle**. In ISC1 versus CG we detected a higher concentration of citrulline and a lower concentration of argininosuccinate: Mutants for argininosuccinate synthase genes have shown a lower virulence than WT parasites.

(iii) **Nucleotide salvage pathway**. This pathway is essential, since *Leishmania* cannot synthesize the purine ring de novo and is therefore dependent on salvaging these from host purines. Our previous results suggested that ISC1 parasites might be better at salvaging nucleotides from their environment.

In a third study, we experimentally demonstrated that ISC1 and CG strains are developing similarly in natural ISC vector *Phlebotomus argentipes*, suggesting that *P. argentipes* is a fully competent vector for ISC1 parasites (*18*).

Altogether, these experimental studies demonstrate differences between ISC1 and CG in antimonial susceptibility and predict major functional differences, including virulence. Taking into account that ISC1 can easily be transmitted by *P. argentipes*, particular attention is required to monitor the fate of ISC1-related population in the region, especially in a post-VL elimination context. Genomic surveillance can clearly be done with the approach here used, but this should be complemented by phenotyping of the new detected *L. donovani* variants.

References

1. Domagalska MA, Imamura H, Sanders M, Van den Broeck F, Bhattarai NR, Vanaerschot M, et al. Genomes of *Leishmania* parasites directly sequenced from patients with visceral leishmaniasis in the Indian subcontinent. Rogers MB, editor. PLoS Negl Trop Dis. 2019 Dec 12; 13(12):e0007900. Available from: https://dx.plos.org/10.1371/journal.pntd.0007900

2. Imamura H, Downing T, Van den Broeck F, Sanders MJ, Rijal S, Sundar S, et al. Evolutionary genomics of epidemic visceral leishmaniasis in the Indian subcontinent. eLife. 2016;5:e12613. PubMed https://doi.org/10.7554/eLife.12613

3. Franssen SU, Durrant C, Stark O, Moser B, Downing T, Imamura H, et al. Global genome diversity of the *Leishmania donovani* complex. eLife. 2020;9:e51243. PubMed https://doi.org/10.7554/eLife.51243

4. Zhang WW, Ramasamy G, McCall LI, Haydock A, Ranasinghe S, Abeygunasekara P, et al. Genetic analysis of *Leishmania donovani* tropism using a naturally attenuated cutaneous strain. PLoS Pathog. 2014;10:e1004244. PubMed https://doi.org/10.1371/journal.ppat.1004244

5. Camacho E, González-de la Fuente S, Rastrojo A, Peiró-Pastor R, Solana JC, Tabera L, et al. Complete assembly of the *Leishmania donovani* (HU3 strain) genome and transcriptome annotation. Sci Rep. 2019;9:6127. PubMed https://doi.org/10.1038/s41598-019-42511-4

6. Li H, Durbin R. Fast and accurate short read alignment with Burrows-Wheeler transform. Bioinformatics. 2009;25:1754–60. PubMed https://doi.org/10.1093/bioinformatics/btp324

7. Danecek P, Bonfield JK, Liddle J, Marshall J, Ohan V, Pollard MO, et al. Twelve years of SAMtools and BCFtools. Gigascience. 2021 Feb 1; 10(2):giab008. Available from: https://pubmed.ncbi.nlm.nih.gov/33590861/

8. McKenna A, Hanna M, Banks E, Sivachenko A, Cibulskis K, Kernytsky A, et al. The Genome Analysis Toolkit: a MapReduce framework for analyzing next-generation DNA sequencing data. Genome Res. 2010;20:1297–303. PubMed https://doi.org/10.1101/gr.107524.110

9. Kozlov AM, Darriba D, Flouri T, Morel B, Stamatakis A. RAxML-NG: a fast, scalable and user-friendly tool for maximum likelihood phylogenetic inference. Bioinformatics. 2019;35:4453–5. PubMed https://doi.org/10.1093/bioinformatics/btz305

10. Yu G. Using ggtree to Visualize Data on Tree-Like Structures. Curr Protoc Bioinformatics. 2020;69:e96. PubMed https://doi.org/10.1002/cpbi.96

11. Huson DH, Bryant D. Application of phylogenetic networks in evolutionary studies. Mol Biol Evol. 2006;23:254–67. PubMed https://doi.org/10.1093/molbev/msj030

12. Potvin JE, Leprohon P, Queffeulou M, Sundar S, Ouellette M. Mutations in an Aquaglyceroporin as a Proven Marker of Antimony Clinical Resistance in the Parasite *Leishmania donovani.* Clin Infect Dis. 2021;72:e526–32. PubMed https://doi.org/10.1093/cid/ciaa1236

13. Dumetz F, Cuypers B, Imamura H, Zander D, D'Haenens E, Maes I, et al. Molecular Preadaptation to Antimony Resistance in *Leishmania donovani* on the Indian Subcontinent. MSphere. 2018;3:e00548–17. PubMed https://doi.org/10.1128/mSphere.00548-17

14. Pountain AW, Weidt SK, Regnault C, Bates PA, Donachie AM, Dickens NJ, et al. Genomic instability at the locus of sterol C24-methyltransferase promotes amphotericin B resistance in *Leishmania* parasites. PLoS Negl Trop Dis. 2019;13:e0007052. PubMed https://doi.org/10.1371/journal.pntd.0007052

15. Pérez-Victoria FJ, Sánchez-Cañete MP, Castanys S, Gamarro F. Phospholipid translocation and miltefosine potency require both *L. donovani* miltefosine transporter and the new protein LdRos3 in *Leishmania* parasites. J Biol Chem. 2006;281:23766–75 . PubMed https://doi.org/10.1074/jbc.M605214200

16. Carnielli JBT, Dave A, Romano A, Forrester S, de Faria PR, Monti-Rocha R, et al. 3'Nucleotidase/nuclease is required for Leishmania infantum clinical isolate susceptibility to miltefosine. EBioMedicine. 2022;86:104378. PubMed https://doi.org/10.1016/j.ebiom.2022.104378

17. Cuypers B, Berg M, Imamura H, Dumetz F, De Muylder G, Domagalska MA, et al. Integrated genomic and metabolomic profiling of ISC1, an emerging *Leishmania donovani* population in the Indian subcontinent. Infect Genet Evol. 2018;62:170–8. PubMed https://doi.org/10.1016/j.meegid.2018.04.021

18. Seblova V, Dujardin JC, Rijal S, Domagalska MA, Volf P. ISC1, a new *Leishmania donovani* population emerging in the Indian sub-continent: Vector competence of *Phlebotomus argentipes.* Infect Genet Evol. 2019;76:104073. PubMed https://doi.org/10.1016/j.meegid.2019.104073

Appendix Table 1. Clinical and epidemiologic data of patients*

**Sample code**	**022**	**023**	**024**
**Sequencing code**	105328–001–022	105328–001–023	105328–001–024
**date_sample_collection**	21/01/2019	24/01/2019	07/02/2019
**District**	Dolpa	Darchula	Bardiya
**Village**	Se-Phoxundo rural Municipality	Juga Rural Municipality	Madhuwan Municipality
**type_disease**	VL	VL	VL
**Past history of VL or PKDL**	No	Past history of VL x 6 Month	No
**past_drug_used**	NA	L-AmB	NA
**current_drug_used**	L-AmB	L-AmB + PMM	L-AmB
**date_treatment_start**	21/01/2019	24/01/2019	07/02/2019
**initial_outcome**	improved	improved	improved
**final_outcome**	no data	cured	cured
**travel_history_VL endemic areas**	traveled history to Surkhet districts	no travel history to VL endemic areas in Nepal & India	History of travel to Uttrakhand, India

*VL, Visceral Leishmaniasis; PKDL, Post Kala Azar Dermal Leishmaniasis; NA, not applicable; L-AmB, Liposomal Amphotericin B; PMM, Paromomycin

Appendix Table 2. Alignment statistics of the three SureSelect processed samples. The estimated percentage of *L. donovani* DNA is the percentage obtained via qPCR analysis as described elsewhere (*1*). The enrichment factor is calculated as the ratio of the percentage of *L. donovani* reads versus the estimated percentage of *L. donovani* DNA obtained via qPCR. The percentage of *L. donovani* with 5x coverage is the percentage of the genome covered with at least 5 reads, and for which SNP detection can be performed.

Sample	Total no. reads	No. reads mapping to *Leishmania donovani*	*L. donovani* DNA, estimated %	*L. donovani* reads, %	Enrichment factor	*L. donovani* with 5× coverage, %
022	51,494,551	5,450,630	0.13	10.58	81	85.06
023	51,591,488	11,615,012	0.16	22.51	141	90.39
024	50,354,911	4,423,258	0.03	8.78	293	85.83

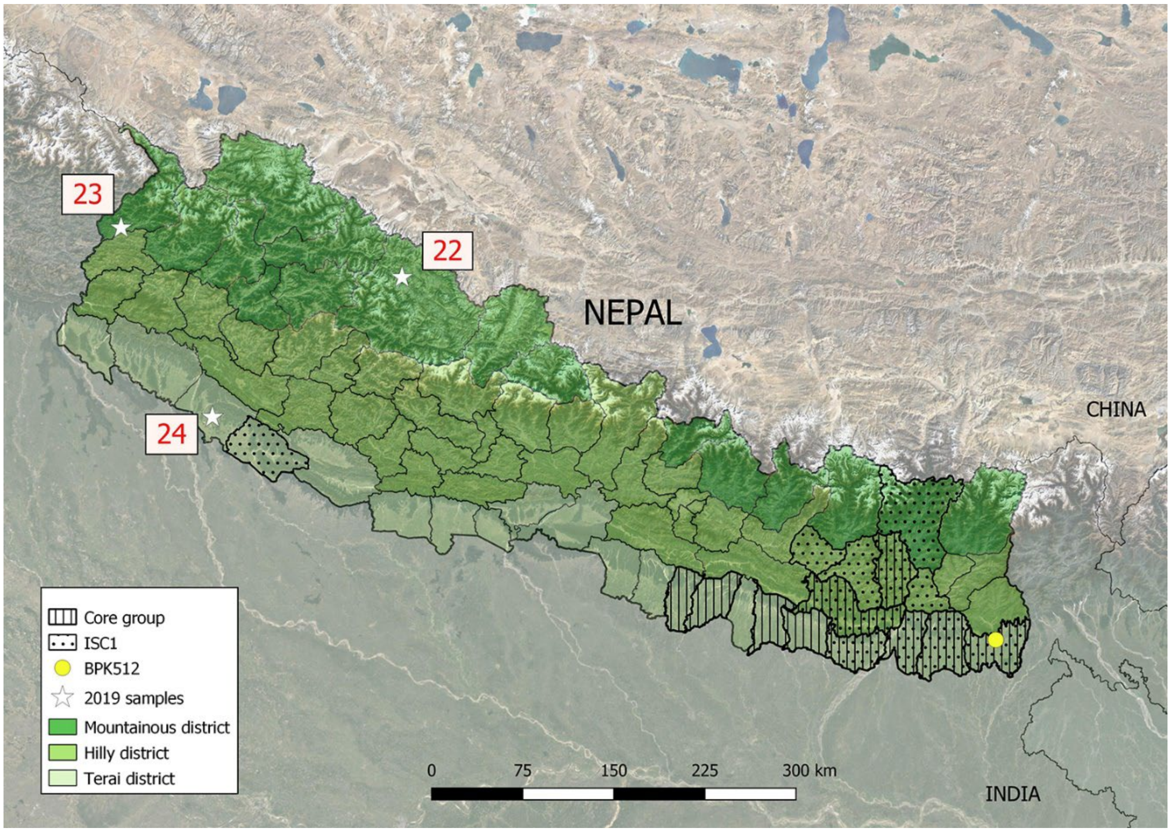

Appendix Figure 1. Geographic origin of the three 2019 samples. The map shows the 77 districts of Nepal and those in which parasites of the core group (vertical hatched) and ISC1 (dotted) were detected from 2000 to 2015. Map was done with qGIS version 3.28.4.

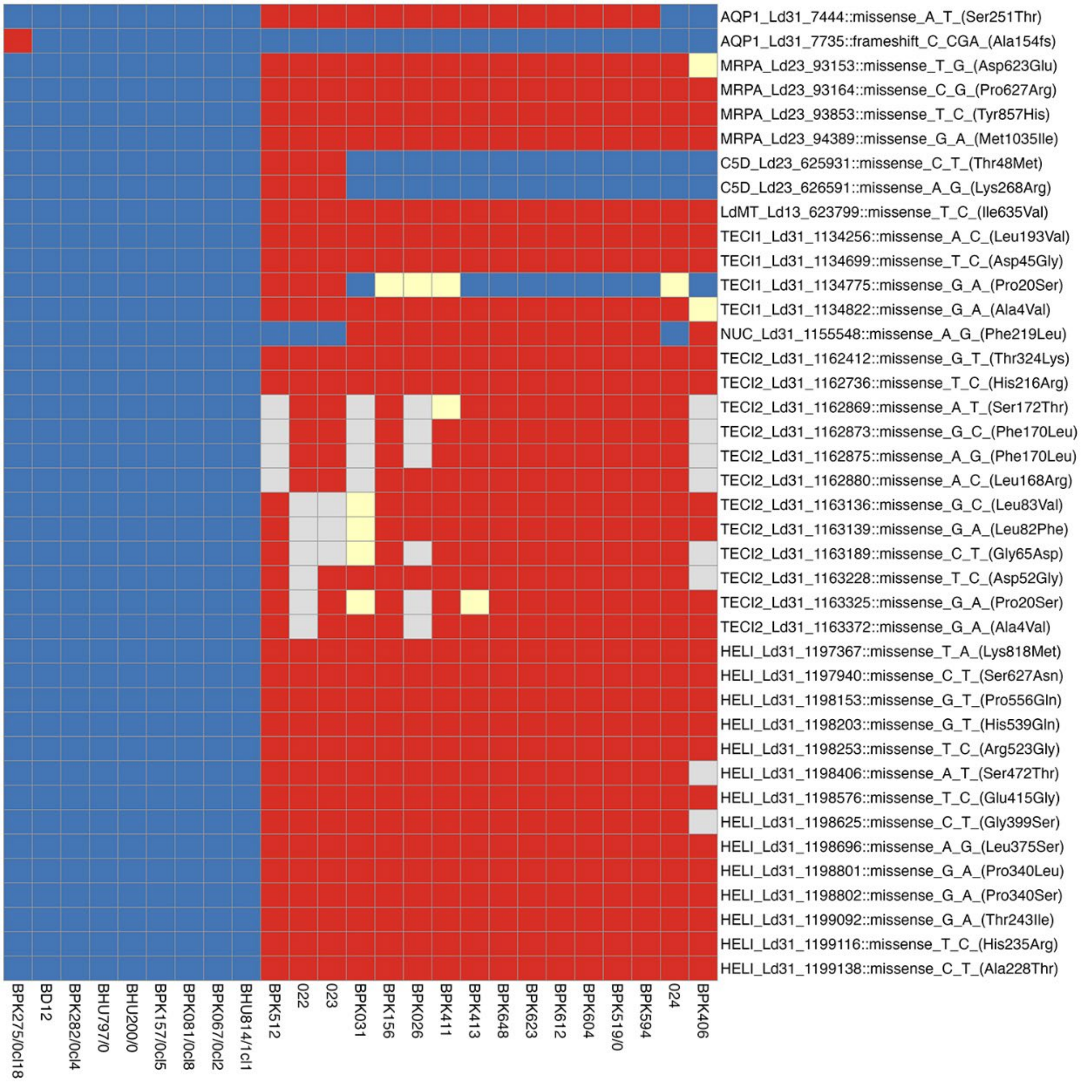

Appendix Figure 2. Heatmap showing the distribution of single nucleotide polymorphisms (SNPs) in genes reported to be associated with drug-resistant phenotypes. The color scheme represents different SNP categories: blue indicates the absence of SNPs, yellow indicates heterozygous SNPs, red indicates homozygous SNPs and gray indicates that not sufficient reads where present to do reliable SNP prediction. The naming convention for SNPs follows the format of the gene of interest, position in the genome, type of mutation, and its effect on the corresponding protein. Samples: BPK512, CG and ISC1 isolates used for the phylogenomic analysis of Figure 1 together with the 3 blood samples of 2019 (022, 023, 024).
